# Modification of Lhx2 activity for *ex vivo* amplification of human iPSC-derived hematopoietic stem/progenitor cells

**DOI:** 10.3389/fcell.2024.1482989

**Published:** 2024-10-15

**Authors:** Kenji Kitajima, Yuna Takahashi, Hikaru Ando, Minako Shingai, Mako Hamasaki, Miyu Tanikawa, Mai Kanokoda, Marino Nakajima, Yasumasa Nishito, Takahiko Hara

**Affiliations:** ^1^ Stem Cell Project, Tokyo Metropolitan Institute of Medical Science, Tokyo, Japan; ^2^ Graduate School of Medical and Dental Sciences, Tokyo Medical and Dental University, Tokyo, Japan; ^3^ Graduate School of Science, Department of Biological Science, Tokyo Metropolitan University, Tokyo, Japan; ^4^ Center for Basic Technology Research, Tokyo Metropolitan Institute of Medical Science, Tokyo, Japan

**Keywords:** iPSCs, HSPCs, organoid, differentiation, Lhx2, homeobox, *ex vivo* amplification

## Abstract

Hematopoietic stem cells (HSCs) obtained from patient-derived human induced pluripotent stem cells (iPSCs) are a promising tool for curing various hematological disorders. We previously demonstrated that enforced expression of the LIM-homeobox transcription factor Lhx2, which is essential for mouse embryonic hematopoiesis, leads to generation of engraftable and expandable hematopoietic stem cells (HSCs) from mouse iPSCs. However, it remained unknown whether Lhx2 can induce HSCs from human iPSCs. Here, we investigated the effect of Lhx2 overexpression on hematopoietic differentiation of human iPSCs. Unexpectedly, Lhx2 severely inhibited proliferation of human iPSC-derived hematopoietic cells. Thus, Lhx2 exhibited differential effects on mouse and human hematopoietic cells. Further studies implied that the inhibitory effect of Lhx2 on human iPSC-derived hematopoietic cells was due to insufficient transcriptional activation ability. Therefore, we modified Lhx2 to strengthen its activity as a transcriptional activator. This modified Lhx2 could induce *ex vivo* amplification of human iPSC-derived hematopoietic stem/progenitor cells (HSPCs). We believe that these findings will facilitate the development of a method to efficiently produce HSCs from human iPSCs.

## 1 Introduction

Hematopoietic stem cells (HSCs) have self-renewal and multi-potential abilities. Taking advantage of this feature, HSCs are used in bone marrow transplantation therapy for malignant hematological diseases (Doulatov et al., 2012). These human HSCs also serve as a source of human immune cells for cancer immunotherapy as well as for basic research of human immunology. For HSC-based therapy, a major concern is that it is difficult to obtain human leukocyte antigen-matched HSCs. Hence, *ex vivo* induction of HSCs from patient-derived induced pluripotent stem cells (iPSCs) is desirable (Demirci et al., 2020). As human iPSCs can give rise to differentiated cells other than hematopoietic lineage, human iPSC-derived HSCs can be transplanted with autologous non-hematopoietic cells, such as hepatocytes, pancreatic β cells, or vascular endothelial cells, into immunocompromised mice. This enables to establish mouse models for research on inflammatory and other diseases involving immune cells (Tran et al., 2020). On the other hand, mouse models carrying human iPSC-derived HSCs could be utilized for research of immune rejection and tolerance, when non-autologous human iPSC-derived cells are transplanted. However, many attempts to generate HSCs from human iPSCs and embryonic stem cells (ESCs) have been failed. Thus, *ex vivo* induction of HSCs from human iPSCs/ESCs remains to be an important challenge in the hematological research field.

Mouse and human HSCs and hematopoietic progenitor cells (HPCs) are enriched in lineage (Lin)^–^Sca-1^+^c-Kit^+^ (LSK) and CD34^+^CD45/43^+^ cell fraction, respectively. Therefore, these cell populations were referred to as hematopoietic stem/progenitor cells (HSPCs). The Lin marker is a cocktail of multiple cell surface markers expressed in differentiated myeloid, erythroid, B, and T cells. We previously demonstrated that enforced expression of the transcription factor Lhx2 induces *ex vivo* amplification of HSPCs from mouse iPSCs/ESCs (Kitajima et al., 2011). Lhx2 was expressed in the embryonic hematopoietic organs such as the yolk sac and aorta/gonad/mesonephros (AGM) region in E10.5 mouse embryos, but not in adult hematopoietic cells (Kitajima et al., 2011). As Lhx2 is essential for fetal liver hematopoiesis in mouse embryos (Porter et al., 1997), it appears to be involved in embryonic but not adult hematopoiesis. In human, LHX2 gene is located on chromosome 9p33 - 34.1 and aberrantly expressed in chronic myelogenous leukemia (CML) (Wu et al., 1996). It was suggested that this aberrant expression is due to conformational changes in the chromatin of LHX2 locus by the chromosomal translocation t (9; 22) (q34; q11) (Wu et al., 1996). However, the relationship between the expression of LHX2 and the leukemogenesis remains unclear.

Lhx2, also known as LH-2/LH2, is a member of LIM-homeobox transcription factor family, and originally isolated from pre-B cells (Xu et al., 1993). It was also identified as a transcription factor that bound to the glycoprotein α-subunit gene promoter (Roberson et al., 1994). Lhx2 is widely expressed in the neuronal lineage. In developing cerebral cortex in mouse embryos, Lhx2 acts as a selector gene for maintaining its regional identity (Mangale et al., 2008). Lhx2 is also involved in the maintenance of mouse hair follicle stem cells (Rhee et al., 2006). Thus, Lhx2 plays important roles in emergence and maintenance of tissue stem cells.

In the case of mouse ESCs, engraftable HSCs were obtained by enforced expression of homeobox transcription factor HoxB4 (Kyba et al., 2002). Earlier than this finding, HoxB4 was known to enhance the induction of HPCs from mouse ESCs (Helgason et al., 1996). The former study used OP9 co-culture, while the latter used embryoid bodies (EBs) for hematopoietic induction of mouse ESCs. Therefore, OP9 co-culture method would be suitable for the induction of HSCs from mouse ESCs. On the other hand, enforced expression of Lhx2 into mouse ESCs resulted in immortalization of HPCs when EB method was used (Pinto do et al., 1998). Therefore, we expected that engraftable HSCs would be obtained by the Lhx2 overexpression when OP9 co-culture was used. As expected, engraftable HSCs were obtained from mouse ESCs and iPSCs by Lhx2 (Kitajima et al., 2011). We also revealed that *ex vivo* amplification of HSPCs from mouse ESCs by Lhx2 was more efficient than that by HoxB4 (Kitajima et al., 2011). Additionally, enforced expression of Lhx2 in adult mouse bone marrow HSCs results in their *ex vivo* amplification (Pinto do et al., 2002; Kitajima et al., 2015). Thus, Lhx2 is an attractive candidate for inducing HSCs from human iPSCs. However, Lhx2 was ineffective for generating engraftable HSCs from human and common marmoset ESCs (Nakajima-Takagi et al., 2013; Kurita et al., 2006). HoxB4 was also unable to induce the engraftable HSCs from human ESCs (Wang et al., 2005). These facts suggest that a road blocker might exist to interfere the function of Lhx2 and HoxB4 in human HSPCs.

In this study, we aimed to achieve *ex vivo* amplification of human iPSC-derived HSPCs by clarifying the inhibitory action of Lhx2 in the human system. By employing a modified Lhx2 (Lhx2TAD) and UM171, a pyrimidoindole derivative that enhances *ex vivo* amplification of human HSPCs (Fares et al., 2014), we succeeded in the expansion of human iPSC-derived HSPCs. This study will be an important step for therapeutic application of human iPSC-derived HSPCs.

## 2 Materials and methods

### 2.1 Establishment, maintenance, and differentiation induction of mouse iPSCs

Mouse iPSCs were reprogrammed from embryonic fibroblasts derived from C57BL6 mice (Nihon SLC) using pMXs-Oct3/4, pMXs-Sox2, pMXs-Klf4, and pMXs-c-Myc (Plasmids #13366, #13367, #13370, and #13375, respectively; Addgene), as previously described (Kitajima et al., 2011). Maintenance and hematopoietic differentiation induction of mouse iPSCs and retroviral transduction of Lhx2 were carried out as previously described (Kitajima et al., 2011). Fluorescence-activated cell sorting (FACS) of hematopoietic cells were performed as previously described (Kitajima et al., 2011). Antibodies used in this study are listed in Supplementary Table S1. Mouse TrueStain FcX (Biolegend) were used to suppress non-specific antibody binding.

### 2.2 Maintenance and differentiation induction of human iPSCs

Human iPSCs (SM28) were used in the first part of this study. They were maintained as previously described (Kitajima et al., 2016). Induction of human iPSCs into hemogenic endothelial cells (HECs) by transient administration of the GSK3β inhibitor CHIR99021 and isolation of HECs were carried out as previously described (Kitajima et al., 2016). The isolated cells were transduced with the retroviral vector and seeded onto OP9 cells with human interleukin (IL)-6 and human stem cell factor (SCF) as previously described (Kitajima et al., 2016). Cell sorting experiments were carried out by using FACSAria™ III (BD biosciences).

Hematopoietic induction of human iPSCs by a new method was carried out as previously described (Kitajima et al., 2022) with minor modifications. Detailed information is provided in the Supplementary Material. Briefly, human iPSCs were treated with CHIR99021 and bone morphogenetic protein four for 2 days. Then, embryoid bodies (EBs) were formed by a forced aggregation method and cultured for an additional 3 days with SB431542 and vascular endothelial growth factor. Thereafter, these EBs were transferred to a tissue culture plate and cultured with various hematopoietic cytokines. In this series of experiments, 692D2 human iPSCs (Okita et al., 2013) were used.

Flow cytometry analyses of hematopoietic cells were performed by using LSRFortessa™ X-20 (BD biosciences) as previously described (Kitajima et al., 2022). Each experiment was performed using 3 wells (*n* = 3), otherwise indicated. Similar results were obtained in independent differentiation induction experiments. Antibodies used in these experiments are listed in Supplementary Table S1. Human TrueStain FcX (Biolegend) was used to suppress non-specific antibody binding. Colony-forming unit (CFU) assays were carried out using H4034 MethoCult medium (Stem Cell Technologies) as previously described (Kitajima et al., 2022).

### 2.3 Transduction of Lhx2 and gene expression analysis in K562 cells

Human K562 cells were cultured in RPMI 1640 medium (Nacalai) supplemented with 10% fetal calf serum and penicillin/streptomycin. Retroviral vectors were prepared using Plat-GP packaging cells as previously described (Miyashita et al., 2018). Two days after transduction, EGFP^+^ cells were isolated and cultured for an additional 3 days. Then, total RNA was prepared using an RNeasy Mini Kit (QIAGEN) and subjected to microarray analysis. For cell proliferation analyses, the retroviral vectors were transduced on day −2. From 2 days later (day 0), the number of EGFP^+^ cells was scored using LSRFortessa™ X-20 (BD Biosciences) every 3 days. Each transduction experiment was performed using 3 wells (*n* = 3) and statistical analyses were carried out. Similar results were obtained in independent transduction experiments.

### 2.4 Retroviral vectors

The retroviral vectors pMY-IRES-EGFP and pMY-FLAG-Lhx2-IRES-EGFP were previously described (Kitajima et al., 2011). Construction of pMY-FLAG-Lhx2TAD-IRES-EGFP is described in the Supplementary Material and Methods. Briefly, the C-terminus of FLAG-Lhx2 was replaced by VP16-TAD via PCR and inserted into pMY-IRES-EGFP.

### 2.5 Reporter assay

The retroviral vectors were used as expression vectors. These vectors were transfected into 293T cells with a reporter plasmid, p6x Lhx2-binding sequence (LBS)-Luc, and pRL-TK using FuGeneHD transfection reagent (Promega) according to the manufacturer’s recommendation. Each transfection experiment was performed using 3 wells (*n* = 3) and statistical analyses were carried out. Similar results were obtained in independent transfection experiments. The luciferase assay was carried out as previously described (Kitajima et al., 2013). Detailed information on the construction of p6x LBS-Luc is provided in the Supplementary Material.

### 2.6 Lentiviral vectors

The lentiviral vectors pHR-IRES-ZsGreen1, pHR-FLAG-Lhx2-IRES-ZsGreen1, and pHR-FLAG-Lhx2TAD-IRES-ZsGreen1 were used. Construction of these vectors is described in the Supplementary Material. Lentiviral vectors were produced as previously described (Kitajima et al., 2022).

### 2.7 Transplantation analyses

Lhx2TAD-transduced cells on day 14 were transplanted into sub-lethally (2.5 Gy) irradiated 20-week-old NOD/scid/γC^−/−^ (NSG) mice (The Jackson Laboratory) by the caudal artery transplantation method (7.5 × 10^5^ cells/mouse; *n* = 4). This method was originally developed to establish a mouse model for the bone metastasis of cancer cells (Kuchimaru et al., 2018). It was recently applied to the transplantation of human HSCs into NSG mice (Sakurai et al., 2023).

To test the efficacy of this method, K562 cells constitutively expressing luciferase gene were prepared by introduction of pLVSIN-EF1a-Luc-IRES-ZsGreen1 lentiviral vector into K562 cells. Then, these cells were transplanted from tail vein or from caudal artery. Transplanted mice were intraperitoneally injected with D-Luciferin (Summit Pharmaceutical International). The imaging analyses were carried out by IVIS Lumina imaging system (PerkinElmer) according to the manufacture’s instruction.

All of animal experimental procedures were pre-approved by the Animal Experimentation Committee at Tokyo Metropolitan Institute of Medical Science.

### 2.8 Statistical analyses

Statistical significance was determined by the two-tailed, paired Student’s t-test using Numbers software (Apple). Mean values with standard deviations are shown in the graphs (*n* = 3). *, **, and *** in the graphs indicate *p* < 0.05, *p* < 0.005, and *p* < 0.0005, respectively.

## 3 Results

### 3.1 Different effects of Lhx2 on hematopoietic differentiation of mouse and human iPSCs

**FIGURE 1:**
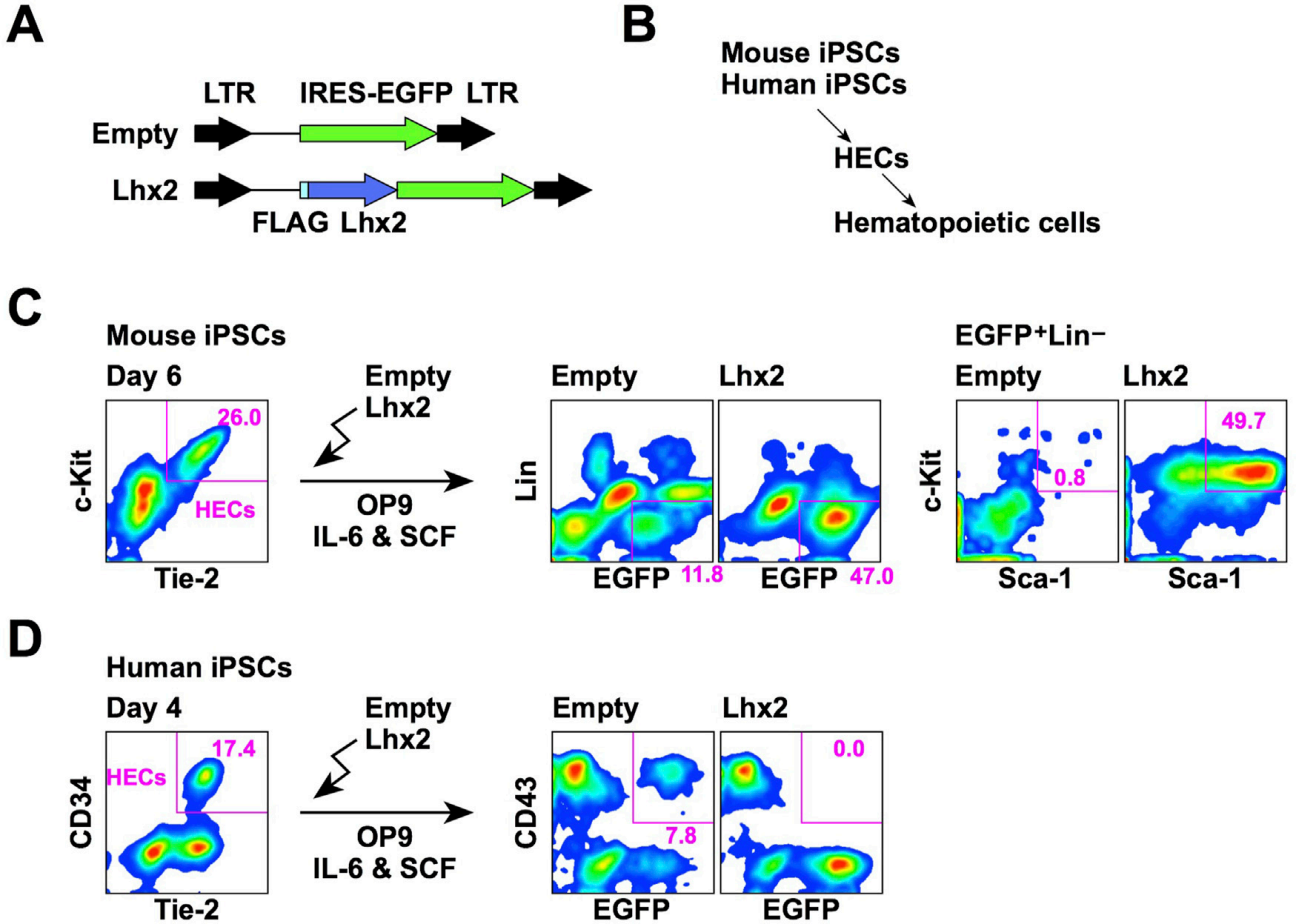
Opposite effects of Lhx2 on hematopoietic induction from mouse and human iPSCs. **(A)** Structure of retroviral vectors used for Lhx2 transduction. **(B)** An overview of hematopoietic differentiation of mouse and human iPSCs. **(C)** Effect of Lhx2 on hematopoietic induction from mouse iPSCs. **(D)** Effect of Lhx2 on hematopoietic induction from human iPSCs. In C and D, the numbers indicate the percentages within the gates. Representative data from multiple experiments are shown.

We first compared the effect of Lhx2 overexpression on hematopoietic cell induction from mouse and human iPSCs. In this series of experiments, a retroviral vector carrying IRES-EGFP was used as the gene delivery tool (Figure 1A). Mouse and human iPSCs were differentiated into hematopoietic cells via HECs (Figure 1B). The HECs are common progenitors for hematopoietic and endothelial cells. In the case of mouse ESCs/iPSCs, HECs are defined as Tie-2^+^c-Kit^+^ cells. When mouse iPSCs were co-cultured with OP9 stromal cells for 6 days, approximately 20%–30% were differentiated into HECs (Figure 1C). To examine an effect of Lhx2, Lhx2 was overexpressed into these HECs and cultured on OP9 cells for 7 days in the presence of mouse IL-6 and mouse SCF. In the case of empty vector, 11.8% of cells were EGFP^+^Lin^–^and among them, Sca-1^+^c-Kit^+^ HSPCs were hardly detected (Figure 1C). On the other hand, when Lhx2 was introduced, 47.0% of cells were EGFP^+^Lin^–^and 49.7% of these cells were Sca-1^+^c-Kit^+^ HSPCs (Figure 1C). Thus, mouse HSPCs were significantly expanded by Lhx2. These data are consistent with our previous study, which also proved that these Lhx2-transduced HSPCs contain engraftable HSCs (Kitajima et al., 2011).

Next, SM28 human iPSCs were differentiated into HECs by our previous protocol using CHIR99021 (Kitajima et al., 2016). In this system, 17% of cells were CD34^+^ on day 4 of differentiation induction (Figure 1D). Our previous study revealed that these CD34^+^ cells are differentiated into hematopoietic and endothelial cells when isolated and co-cultured with OP9 (Kitajima et al., 2016). Furthermore, all of these CD34^+^ cells expressed TIE-2 (Figure 1D), a marker of HECs. Therefore, these cells were considered to be HECs. However, the human ESC-derived CD34^+^ cells could differentiate into mesenchymal stem cells (Kopher et al., 2010). Although mouse and human HECs possessed the hemogenic activity, mouse and human HECs might have different differentiation potentials.

To analyze the effects of Lhx2 on hematopoietic induction of human iPSCs, HECs were transduced with Lhx2 and cultured on OP9 cells in the presence of human IL-6 and human SCF. In the case of human ESCs/iPSCs, CD43 is widely used as an earliest marker of hematopoietic cells (Vodyanik et al., 2006). An authentic marker of pan-hematopoietic cells, CD45, is known to be expressed later. When the empty vector was transduced, 7.8% were EGFP^+^CD43^+^ (Figure 1D). By contrast, EGFP^+^CD43^+^ cells were not detected upon Lhx2 transduction (Figure 1D). This was due to the inhibitory effect of Lhx2 on growth of human iPSC-derived hematopoietic cells, as is shown later. Thus, in sharp contrast to mouse iPSCs, Lhx2 could not induce HSPCs from human iPSCs.

### 3.2 Growth inhibition of K562 cells by Lhx2

**FIGURE 2:**
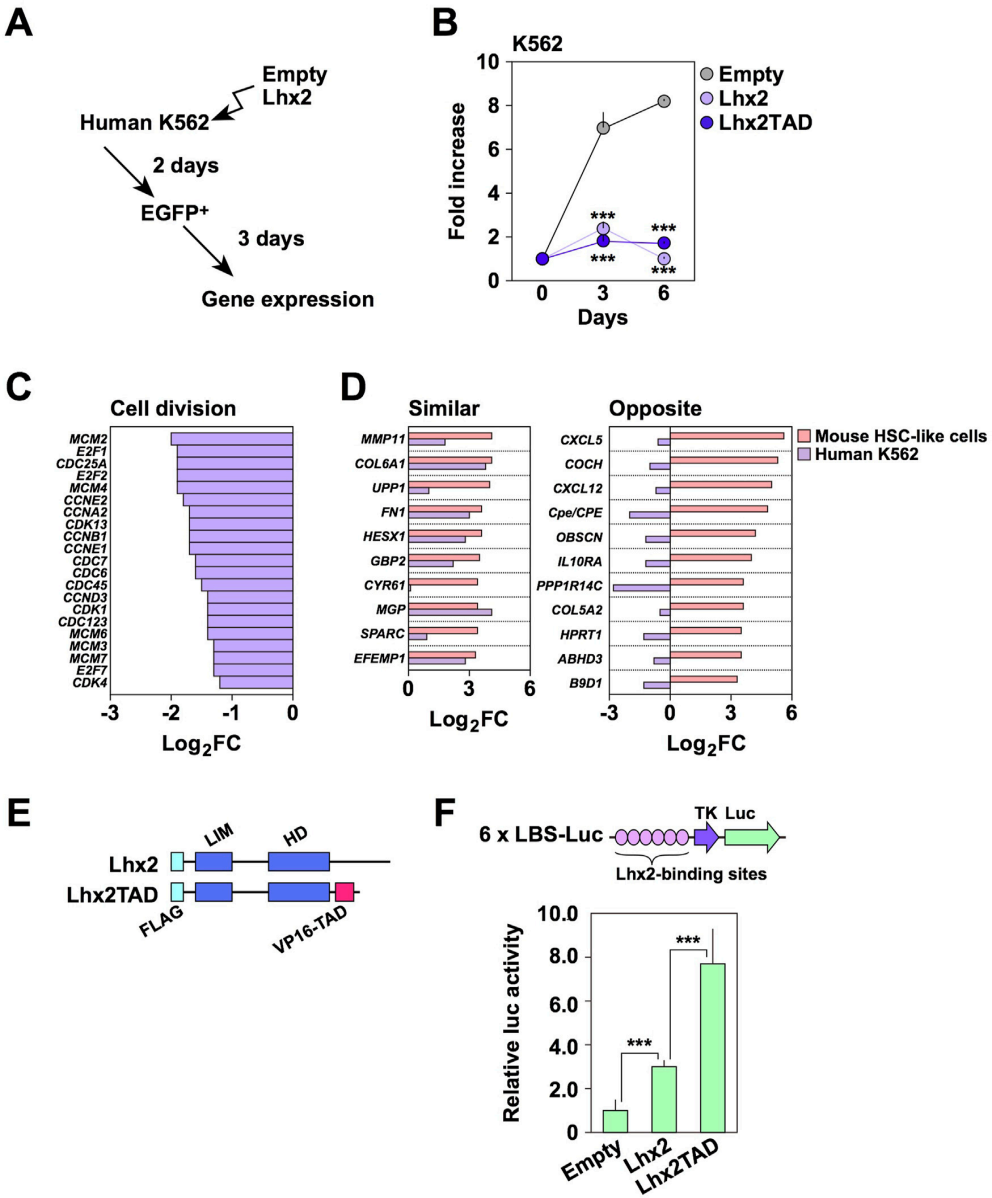
Effect of Lhx2 on gene expression in K562 cells. **(A)** An overview of Lhx2 transduction experiments. Lhx2 was transduced with the retroviral vector as shown in Figure 1A. **(B)** Suppression of proliferation of K562 cells by Lhx2 and Lhx2TAD. The vertical axis indicates the relative cell increase rates compared with the number of cells on day 0. Mean values with standard deviations are shown (*n* = 3). ***p < 0.0005 by the Student’s *t*-test compared with empty vector-transduced cells. **(C)** Effect of Lhx2 on expression of cell division-related genes in K562 cells. **(D)** Effect of Lhx2 on expression of genes in K562 cells whose expression was increased by Lhx2 in mouse HSPCs (Lhx2-activated genes). In C and D, the horizontal axis indicates the Log2 expression levels of genes in Lhx2-transduced cells compared with empty vector-transduced cells. **(E)** Structure of Lhx2 and Lhx2TAD. **(F)** Transcriptional activation ability of Lhx2 and Lhx2TAD. The results of a reporter assay using 6x LBS-Luc are shown. The values were corrected by co-transfected RL-TK. Mean values with standard deviations are shown (*n* = 3). ***p < 0.0005 by the Student’s *t*-test.

To obtain clues about the inhibitory effect of Lhx2 in human cells, we used human leukemia cell lines, since we previously found that Lhx2 inhibits the proliferation of human T-cell leukemia cell lines, Jurkat and CCRF-CEM (Miyashita et al., 2018). To this end, we selected a human CML cell line, K562, since CML is derived from HSCs. In this cell line, endogenous LHX2 expression was not detected (data not shown), although the previous study indicated human CML expressed LHX2 (Wu et al., 1996). K562 cells were transduced with empty vector or Lhx2 expression vector (Figure 2A). When empty vector was transduced, EGFP^+^ cells increased 7.0 ± 0.7 folds and became confluent on day 3 (Figure 2B). In contrast, Lhx2-transduced EGFP^+^ cells only increased 2.4 ± 0.3 and 1.7 ± 0.2 folds on day 3 and 6, respectively (Figure 2B). There as a statistically significant difference between empty- and Lhx2 vector-transduced cells on day 3 (*p* = 0.0002) and on day 6 (*p* = 7.4 × 10^−8^) (*n* = 3). Thus, Lhx2 overexpression severely suppressed the proliferation of K562 cells.

We next analyzed whether transcriptional regulatory functions of Lhx2 in K562 cells are similar to those in mouse HSPCs or not. We previously identified genes whose expression levels were increased by Lhx2 overexpression in mouse ESC-derived HSPCs (Kitajima et al., 2013). We hereafter refer to these genes as “Lhx2-activated genes”. Based on this data, we investigated whether the transcription of these genes is altered by Lhx2 in K562 cells using microarray analyses with empty vector- and Lhx2-transduced K562 cells. As expected, Lhx2 decreased the expression of cell division-related genes (Figure 2C). In addition, Lhx2 acted as a transcriptional repressor of erythroid and platelet-related genes in K562 cells (Supplementary Figure S1).

Next, we analyzed the expression of Lhx2-activated genes in K562 cells. Among the top 50 Lhx2-activated genes, 21 genes were expressed in K562 cells. Among these 21 genes, expression of 10 genes was increased while expression of the other 11 genes was decreased by Lhx2 overexpression in K562 cells (Figure 2D). Lhx2 oppositely regulated the transcription of 11 out of 21 Lhx2-activated genes in K562 cells. Based on this finding, we speculated that the inhibitory effect of Lhx2 on the proliferation of human hematopoietic cells is related to its transcriptional repressor activity.

If transcriptional activator function was forcibly provided to Lhx2, its transcriptional repressor activity would be attenuated. To investigate this possibility, transcriptional activation domain (TAD) of VP16 was connected to the C-terminal region of Lhx2 (Figure 2E), since addition of TAD to several transcription factors was known to enhance their biological functions. For example, reprogramming of somatic cells into iPSCs was enhanced by OCT4 with TAD (Hirai et al., 2012). In hematopoietic lineage, induction of myeloid leukemia by oncogenic homeobox transcription factor Meis1 was promoted by the addition of TAD (Wang et al., 2006). As TAD itself has no DNA binding ability, it is unlikely that TAD directly activates transcription of unrelated genes.

The Lhx2 and TAD fusion chimera, Lhx2TAD, exhibited higher transcriptional activity than wild-type Lhx2 (Figure 2F). When compared with empty vector, Lhx2 and Lhx2TAD vectors increased the reporter activity by 3.0 ± 0.3 and 7.0 ± 1.6 folds, respectively. Lhx2TAD exhibited 2.6 ± 0.5 times higher activity than Lhx2 (Figure 2F). These differences were statistically significant (*p* = 0.001, *n* = 3). Next, effects of Lhx2 and Lhx2TAD on the growth of human K562 cells were compared. However, growth of both Lhx2-and Lhx2TAD-transduced K562 cells was inhibited (Figure 2B). Lhx2TAD-transduced EGFP^+^ cells only increased 1.0 ± 0.1 and 1.7 ± 0.1 folds on day 3 and 6, respectively (Figure 2B). The difference between empty vector and Lhx2TAD vector was statistically significant on day 3 (*p* = 0.0002) and on day 6 (*p* = 3.1 × 10^−8^) (*n* = 3). Thus, the repressive activity of Lhx2 in K562 cells was not reversed by the addition of TAD.

### 3.3 Induction of hematopoietic cells from human iPSCs

**FIGURE 3:**
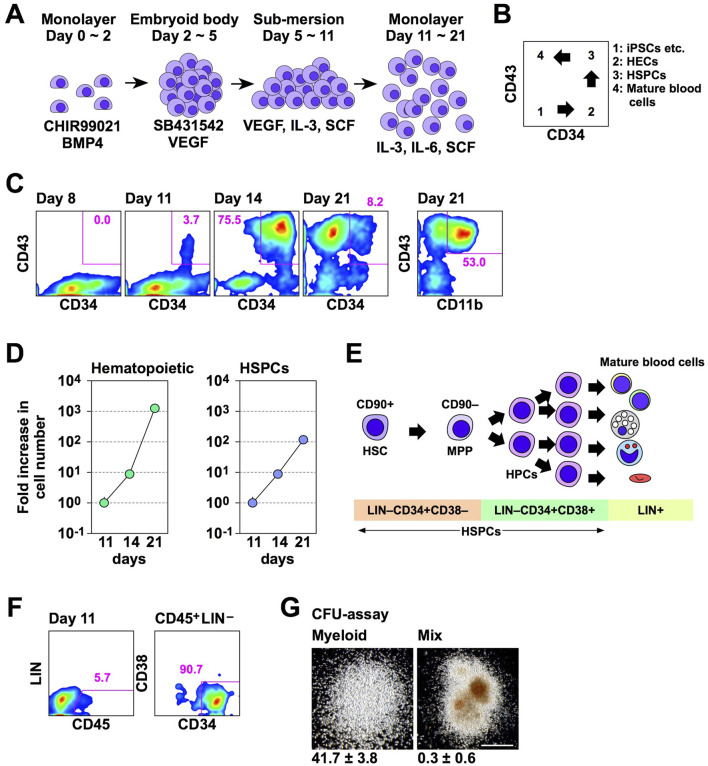
A novel hematopoietic induction system for human iPSCs. **(A)** Schematic outline of HSPC induction from human iPSCs. **(B)** Schematic diagram of flow cytometer analysis of hematopoietic differentiation from human iPSCs. **(C)** Time course of hematopoietic cell differentiation. **(D)** Increase in the number of hematopoietic cells (CD43^+^) and HSPCs (CD43^+^CD34^+^) during differentiation induction. The vertical axis indicates the relative cell increase rates compared with the number of cells on day 11. Mean values with standard deviations are shown (*n* = 3). **(E)** Representative cell surface markers at each differentiation stage of human HSPCs. MPP; multi-potent progenitor. **(F)** HSPC marker expression on day 11. In C and D, numbers indicate percentages within the gates. Representative data from multiple experiments are shown. **(G)** CFU assay of CD45^+^LIN^–^CD34^+^CD38^–^ cells on day 11. Representative myeloid and mix colonies are shown on the left and right, respectively. Scale bar = 500 µm. The numbers at the bottom of images indicate the average number of colonies obtained from 200 cells with standard deviations (*n* = 3).

Before examining the effect of Lhx2 and Lhx2TAD on human iPSC-derived hematopoietic cells, we evaluated efficacy and kinetics of our new differentiation induction system of human iPSCs into HSPCs using non-transduced iPSCs. In a series of experiments, 692D2 human iPSCs were used since this iPSC line is more widely used cell line than SM28. An overview of our new induction method is shown in Figure 3A. Differentiation of human iPSCs into the hematopoietic cells progresses as follows; CD34^–^CD43^–^ (iPSCs), CD34^+^CD43^–^ (HECs), CD34^+^CD43^+^ (HSPCs), and CD34^–^CD43^+^ (mature blood cells), as illustrated in Figure 3B.

In experiments with this protocol, HSPCs emerged and their number increased on days 11 and 14 of differentiation induction, respectively (Figure 3C). Subsequently, most hematopoietic cells were differentiated into mature blood cells on day 21 (Figure 3C). Percentages of HSPCs on days 8, 11, 14, and 21 were 0.0, 3.7, 75.5, and 8.2, respectively (Figure 3C). On day 21, 53.0% of hematopoietic cells were differentiated to CD11b/Mac-1^+^ myeloid cells (Figure 3C). The number of hematopoietic cells exponentially increased between days 11 and 21 of the hematopoietic cell induction culture (Figure 3D). The number of HSPCs also increased, but at a slower speed than overall hematopoietic cells (Figure 3D). The number of hematopoietic cells were increased 7.9 ± 0.1 and 1,330.4 ± 83.5 folds on days 14 and 21, respectively, while HSPCs were increased 7.4 ± 0.2 and 115.0 ± 11.7 folds on days 14 and 21, respectively, when compared with that of corresponding cells on day 11 (Figure 3D). Consequently, the percentage of HSPCs was decreased on day 21 (Figure 3C). Thus, in this culture condition, the number of human iPSC-derived HSPCs increased between days 11 and 21, and a large number of differentiated progenies were produced thereafter.

A simplified overview of adult human hematopoiesis in bone marrow is illustrated in Figure 3E (Doulatov et al., 2012). To clarify which stages of cells were first induced from human iPSCs, we analyzed the expression of CD45, lineage markers, and CD38. Consequently, CD45^+^Lin^–^CD34^+^CD38^–^ cells emerged on day 11 (Figure 3F). When these cells were isolated and cultured in methyl-cellulose culture medium containing SCF, IL-3, erythropoietin (EPO), granulocyte colony-stimulating factor (G-CSF), and granulocyte-macrophage colony-stimulating factor (GM-CSF) for 2 weeks, myeloid and mix colonies were observed (Figure 3G). Thus, multi-potent HSPCs were induced from human iPSCs in this culture system. However, when 200 cells were cultured, 41.7 ± 3.8 myeloid colonies emerged, while mix colonies were 0.3 ± 0.6 (Figure 3G). Thus, more than 90% of colonies were myeloid type. Related to this finding, this culture condition mainly gave rise to myeloid cells on day 21 (Figure 3C). Therefore, these HSPCs have myeloid-biased differentiation potential.

### 3.4 Effects of Lhx2 and Lhx2TAD on hematopoietic cell induction from human iPSCs

**FIGURE 4:**
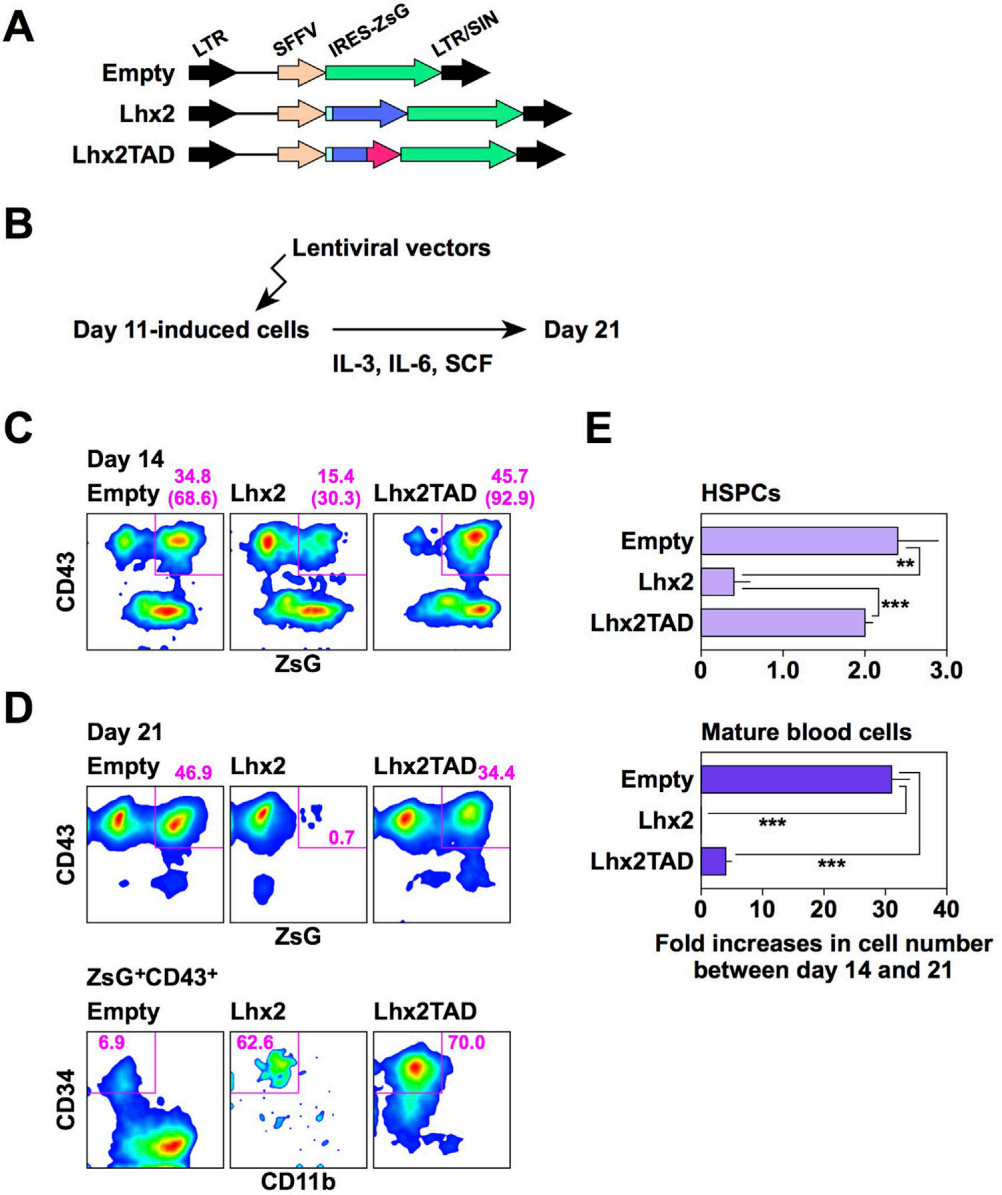
Effects of Lhx2 and Lhx2TAD on hematopoietic induction of human iPSCs. **(A)** Structure of lentiviral vectors used for Lhx2 transduction. ZsG = ZsGreen1. **(B)** Diagram of the lentiviral transduction schedule for human iPSC-derived differentiated cells. **(C, D)** FACS analyses on days 14 and 21. The numbers indicate the percentages within the gates. Representative data from multiple experiments are shown. In C, the numbers in parentheses are the percentage of ZsG^+^ cells among CD43^+^ cells. **(E)** The increase rate of the cell number between days 14 and 21. The horizontal axis indicates the relative cell increase rates on day 21 compared with the number of cells on day 14. Mean values with standard deviations are shown (*n* = 3). **p < 0.005 and ***p < 0.0005 by the Student’s *t*-test.

We next examined the effects of Lhx2 and Lhx2TAD on hematopoietic cell differentiation from human iPSCs using the new induction method. A lentiviral vector (Figure 4A) was used for gene transduction because the retroviral vector was less efficient in this experimental setting. Human iPSCs were induced to differentiate, and lentiviral vectors (empty, Lhx2 and Lhx2TAD) were transduced on day 11 of induction. Then, these cells were cultured with IL-3, IL-6, and SCF (Figure 4B). In this system, on day 14, the transduced cells became ZsGreen1^+^, while non-transduced cells were ZsGreen1^–^.

When Lhx2 was transduced, the percentage of ZsGreen1^+^ cells significantly decreased from 15.4% to 0.7% on days 14 and 21, respectively (Figures 4C, D). In contrast, the percentages of empty vector-transduced ZsGreen1^+^ hematopoietic cells were 34.8% and 46.9% on days 14 and 21, respectively (Figures 4C, D). Thus, growth of Lhx2-transduced hematopoietic cells was significantly suppressed. Notably, 62.6% of Lhx2-transuced hematopoietic cells on day 21 were HSPCs, whereas only 6.9% were HSPCs and myeloid cells were the major population in the case of empty vector-transduction (Figure 4D). Thus, Lhx2 could maintain the un-differentiated state of human iPSC-derived HSPCs, but could not expand them.

Next, the impact of Lhx2TAD overexpression was analyzed. Remarkably, the percentage of Lhx2TAD-transduced hematopoietic cells was drastically increased on days 14 and 21 of differentiation induction, as compared with the case of Lhx2 (Figures 4C, D). The percentages of Lhx2TAD-transduced hematopoietic cells on days 14 and 21 were 45.7% and 34.4%, respectively (Figures 4C, D). Furthermore, the vast majority of Lhx2TAD-transduced hematopoietic cells were HSPCs on day 21 (Figure 4D). Lhx2 overexpression decreased the number of HSPCs between days 14 and 21, whereas such an inhibitory effect was not observed in Lhx2TAD-transduced HSPCs (Figure 4E). Between days 14 and 21, the number of HSPCs transduced with empty vector or Lhx2TAD was increased 2.8 ± 0.5 or 2.4 ± 0.1 folds, respectively, while the number of Lhx2-transuduced HSPCs was decreased to 0.5 ± 0.4 folds (Figure 4E). The differences between empty and Lhx2, and Lhx2 and Lhx2TAD were statistically significant, *p* = 0.0065 and 0.0016, respectively (*n* = 3).

When empty vector was transduced, the number of mature blood cells increased 31.2 ± 2.7 folds (Figure 4E). On the other hand, cells transduced with Lhx2 or Lhx2TAD did not efficiently increase, 0.4 ± 0.2 (*p* = 3.6 × 10^−5^) and 3.9 ± 0.7 folds (*p* = 3.8 × 10^−5^), respectively, as compared with empty vector-transduced cells (*n* = 3) (Figure 4E). Thus, Lhx2 and Lhx2TAD overexpression repressed production of mature blood cells, as compared with empty vector.

### 3.5 Characteristics of Lhx2TAD-transduced human iPSC-derived HSPCs

**FIGURE 5:**
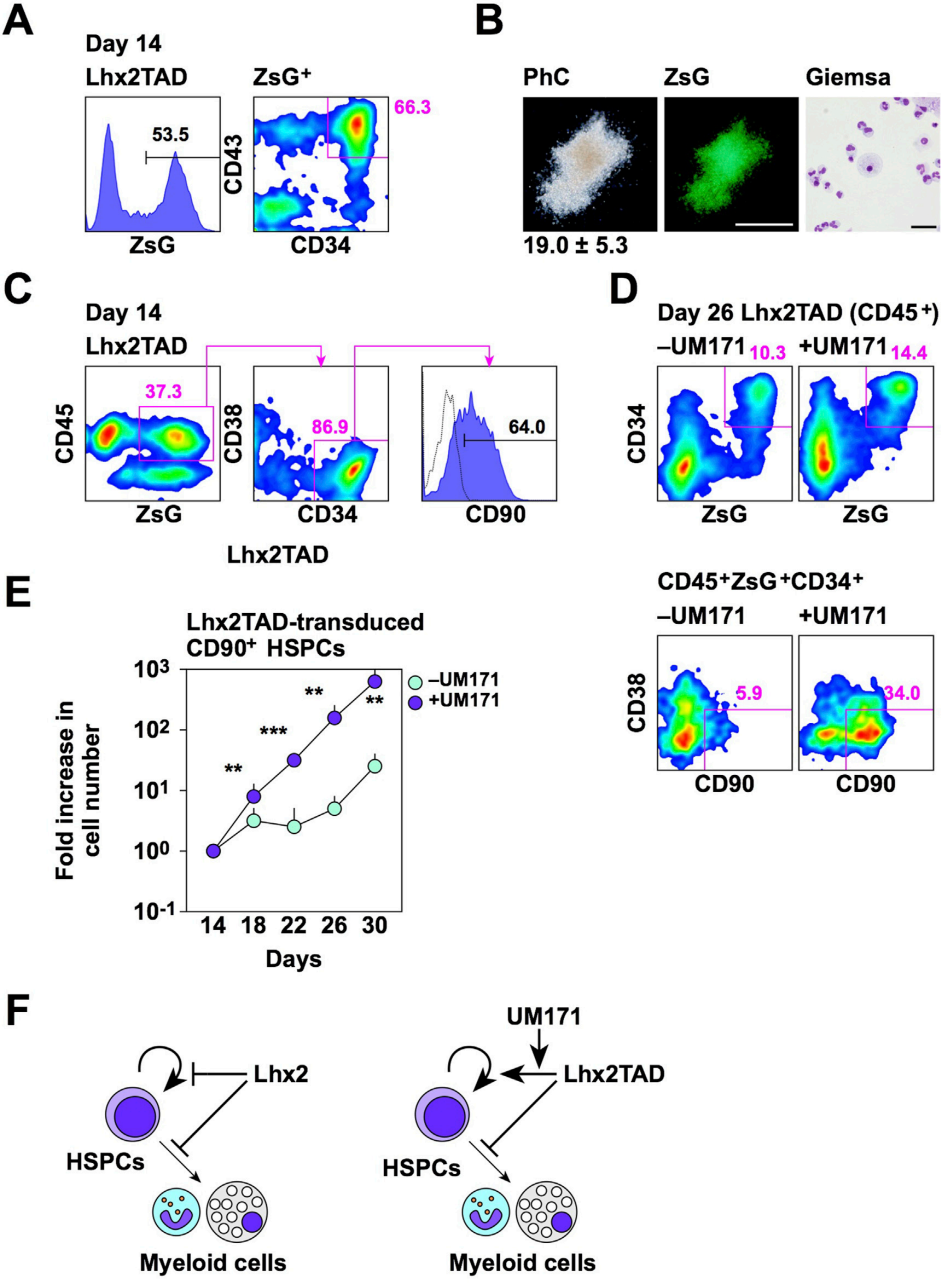
Properties of Lhx2TAD-transduced CD43^+^CD34^+^ cells. **(A)** FACS analysis of Lhx2TAD-transduced cells on day 14. **(B)** CFU assay of Lhx2TAD-transduced ZsG^+^CD43^+^CD34^+^ cells on day 14. The gross morphology of a representative hematopoietic colony and its ZsG expression are shown in the left and middle images, respectively. Scale bar = 500 µm. May-Grünwald/Giemsa staining of cells obtained from this colony is shown on the right. Scale bar = 50 µm. The number at the bottom of the left image indicates the average number of colonies obtained from 200 cells with the standard deviation (*n* = 3). **(C)** HSPC marker expression of Lhx2TAD-transduced ZsG^+^ cells on day 14. **(D)** Effect of UM171. HSPC marker expression was analyzed on day 22. In A, C, and D, the numbers indicate the percentages within the gates. Representative data from multiple experiments are shown. **(E)** The increase rate of the number of ZsG^+^CD45^+^CD34^+^CD38^–^CD90^+^ cells. The horizontal axis indicates the relative cell increase rates compared with the number of cells on day 14. Mean values with standard deviations are shown (*n* = 3). *p < 0.05, **p < 0.005, and ***p < 0.0005 by the Student’s *t*-test. **(F)** Schematic model of the effects of Lhx2 and Lhx2TAD on proliferation and differentiation of human iPSC-derived HSPCs.

To clarify the functional properties of Lhx2TAD-transduced HSPCs, several experiments were carried out. First, we examined whether Lhx2TAD-transduced HSPCs could produce mature hematopoietic cells. Lhx2TAD was transduced on day 11 and ZsGreen1^+^ HSPCs were isolated on day 14 (Figure 5A). On day 14, 53.5% of cells were ZsGreen1^+^ and 66.3% of these cells were HSPCs (Figure 5A). Then, these cells were cultured in methyl-cellulose culture medium containing SCF, IL-3, EPO, G-CSF, and GM-CSF for 2 weeks. Consequently, Lhx2TAD-transduced HSPCs gave rise to ZsGreen1^+^ hematopoietic colonies, which were mostly composed of neutrophils and macrophages (Figure 5B). When 200 cells were inoculated, 19.0 ± 5.3 colonies were formed and all of these colonies were myeloid-type. Thus, Lhx2TAD did not significantly affect cytokine-induced myeloid differentiation. On the other hand, mix colonies were not observed. It remains unclear whether Lhx2TAD-transduced HSPCs were myeloid-committed HPCs. Next, authentic HSPC markers were analyzed. In human HSPCs, most undifferentiated cell population is CD90^+^ (Figure 3E). When Lhx2TAD was transduced, 37.3% of cells were ZsGreen1^+^CD45^+^, 86.9% of these cells were CD34^+^CD38^–^, and 64.0% of ZsGreen1^+^CD45^+^ CD34^+^CD38^–^ cells were CD90^+^ on day 14 (Figure 5C). Therefore, based on the cell surface marker expression patterns, Lhx2TAD-transduced HSPCs could be recognized as HSCs and/or multi-potent HPCs.

As Lhx2-transduced HSPCs contained CD90^+^ cells, transplantation analyses were carried out. NOD/SCID immunocompromised mice lack B and T cells. Thereby human cells are engraftable into these mice. NSG mice are NOD/SCID mice lacking common γ chains of cytokine receptors for IL-2, IL-4, IL-7, IL-9, IL-15, and IL-21. Therefore, in addition to B/T cell deficiencies, NK cells are also deficient in NSG mice in which human cells are highly engraftable. Recently, a new transplantation protocol was developed to increase transplantation efficiency. That is injection of donor cells into caudal artery of mouse tail allowing the delivery of donor cells near the femur without passing through lung. In preliminary experiments using K562 cells expressing luciferase, donor cells were found near the femur when transplanted via caudal artery (Supplementary Figure S2A left). In contrast, intravenous transplantation of donor cells, frequently used in previous transplantation studies, resulted in the accumulation of donor cells in lung or heart 1–2 h after the transplantation (Supplementary Figure S2A right). Since caudal artery transplantation was easier than intra-bone marrow transplantation, we used this transplantation procedure. Lhx2TAD-transduced cells on day 14 were transplanted into conditioned NSG mice. However, donor-derived human hematopoietic cells were hardly detected in peripheral blood and bone marrow of recipient mice at 16 weeks after transplantation (Supplementary Figure S2B, C). Thus, Lhx2TAD overexpression did not confer repopulating activity to human iPSC-derived HSPCs.

Although Lhx2TAD could expand HSPCs when compared to Lhx2, Lhx2TAD-transduced HSPCs only increased 2.1-fold between days 14 and 21 (Figure 4E). Furthermore, Lhx2TAD-transduced HSPCs, as well as empty vector-transduced HSPCs, on day 21 were no longer forming hematopoietic colonies as revealed by colony assays. Therefore, this culture condition is not suitable for *ex vivo* amplification of HSPCs. We previously found that UM171 is effective for maintaining the un-differentiated state of human iPSC-derived HSPCs (Kitajima et al., 2022). Therefore, we cultured Lhx2TAD-transduced cells in the presence of UM171 from day 14. As expected, UM171 retained Lhx2TAD-transduced CD90^+^ HSPCs on day 26 (Figure 5D). CD90^+^ cells were most immature cells in human HSPCs (Figure 3E). When Lhx2TAD was transduced, 10.3% of hematopoietic cells were ZsGreen1^+^ HSPCs and 5.9% of these cells were CD90^+^ in the absence of UM171, while 14.4% of hematopoietic cells were ZsGreen1^+^ HSPCs and 34.0% of these cells were CD90^+^ in the presence of UM171 (Figure 5D). The number of Lhx2TAD-transduced CD90^+^ HSPCs increased 644.2 ± 117.6 and 22.7 ± 7.6 folds when cultured with and without UM171, respectively, between days 14 and 30 (Figure 5E). This difference was statistically significant (*p* = 0.0008, *n* = 3). Thus, Lhx2TAD-transduced HSPCs could be efficiently expanded using UM171.

## 4 Discussion

Here, we demonstrated that enforced expression of Lhx2 suppressed proliferation of human iPSC-derived HSPCs. On the other hand, it induced engraftable HSCs from mouse iPSCs (Kitajima et al., 2011). Thus, Lhx2 exhibits differential effects on hematopoietic cell induction from human and mouse iPSCs. Differences in the properties of human and mouse cells are well known in the case of ESCs/iPSCs. Mouse and human ESCs/iPSCs are closer to the cells of inner cell mass and epiblasts, respectively, in developing embryos. In addition, their cytokine requirements for keeping undifferentiated states are different. On the other hand, little is known about differences between mouse and human HSCs. As described above, HoxB4 was unable to induce HSCs from human ESCs (Wang et al., 2005). Therefore, our findings presented here will be important information for translating mouse study to human system.

Lhx2 suppressed expression of several genes in human K562 cells whose expression was upregulated by Lhx2 in mouse HSPCs. Based on this finding, we speculate that Lhx2 oppositely regulates expression of genes involved in *ex vivo* amplification of HSPCs in mouse and human HSPCs. Lhx2 binds to MRG1 and functions as a transcriptional activator (Glenn and Maurer, 1999). It can also associate with an E3 ubiquitin ligase RLIM, which recruits the Sin3A/histone deacetylase co-repressor complex (Bach et al., 1999). Interactions between Lhx2 and these factors might differ between human and mouse HSPCs.

K562 cells gave us a valuable clue for the inhibitory effect of Lhx2 on growth of human HSPCs. However, not all of the experimental results using K562 cells were applied to human HSPCs. Accessibility of transcription factors to their target genes is largely determined by cell type-specific epigenetic pattern. Probably, only a part of genes is commonly regulated by Lhx2 between K562 cells and human HSPCs. We found that Lhx2TAD suppressed the growth of human K562 cells. This data suggested that although K562 cells was initially considered as a model for human HSPCs, these cells might have properties similar to mature blood cells. Probably, the inhibitory effect of Lhx2TAD on growth of K562 cells is related to its inhibitory effect on the production of mature blood cells from human iPSC-derived HSPCs. Lhx2TAD might suppress the growth of human K562 cells via a transcriptional activation-independent mechanism. We previously found that Lhx2 disrupts the Ldb1:Lmo2 transcription factor complex via its LIM domain (Kitajima et al., 2013). Therefore, destruction of the Ldb1:Lmo2 complex by Lhx2TAD might inhibit the growth of K562 cells.

Our flow cytometer analyses revealed that Lhx2TAD-transduced human iPSC-derived HSPCs included CD90^+^ cells. However, the colony assays revealed that only myeloid colonies were formed by these HSPCs. Therefore, it is necessary to consider which stage of cells are contained in Lhx2TAD-transduced HSPCs. It remains unknown whether these HSPCs could differentiate into other lineages of hematopoietic cells or not. In the case of mouse ESCs/iPSCs, Lhx2-induced engraftable HSCs failed to differentiate into T cells (Kitajima et al., 2011). This is because Lhx2 severely suppresses T cell differentiation. In fact, T cell differentiation potential of the Lhx2-induced mouse HSCs was proved by using doxycycline-mediated conditional gene expression system for Lhx2 expression (Kitajima et al., 2013). Therefore, we could evaluate the differentiation capacity of Lhx2TAD-transduced human iPSC-derived HSPCs by employing a conditional gene expression system.

Lhx2TAD-induced human iPSC-derived HSPCs did not exhibit *in vivo* hematopoietic repopulation potential. We found that IL-3 is required for *in vitro* cell proliferation of HSPCs obtained with Lhx2TAD (Supplementary Figure S3). Therefore, one plausible explanation for this failure is the lack of cross-reactivity of IL-3 and GM-CSF between human and mouse. Genetically engineered NSG mice expressing human IL-3 and GM-CSF might be suitable recipients to detect hematopoietic repopulation activity of Lhx2TAD-transduced human iPSC-derived HSPCs.

Co-introduction of 3 transcription factors (HOXA9, RORA and ERG) confers the self-renewal ability on human iPSC-derived HSPCs (Doulatov et al., 2013). Therefore, comparative analyses of Lhx2TAD-transduced HSPCs with these HSPCs would provide useful information for molecular mechanisms underlying self-renewal of human HSPCs. Interestingly, co-introduction of additional transcription factors, SOX4 and MYB, in addition to above 3 transcription factors, induced engraftable HSPCs from human iPSCs (Doulatov et al., 2013). Therefore, engraftable HSCs might be obtained by introducing other transcription factors into Lhx2TAD-transduced human iPSC-derived HSPCs.

We showed that *ex vivo* amplification of Lhx2TAD-transduced human iPSC-derived HSPCs is supported by UM171. UM171 is known to inhibit the activity of a transcriptional repressor complex, CoREST, containing histone H3K4 demethylase LSD1 (Subramaniam et al., 2020; Chagraoui et al., 2021). Therefore, the supportive effect of UM171 on *ex vivo* amplification of Lhx2TAD-transduced HSPCs was mediated through the inhibition of histone H3K4 demethylation. These notions suggested that histone H3K4 methyl-transferases, such as MLL genes, are interesting candidates for generating engraftable HSCs from Lhx2TAD-transduced human iPSC-derived HSPCs. In addition, comparative gene expression analyses of empty vector, Lhx2, and Lhx2TAD-transduced human iPSC-derived HSPCs could identify genes pivotal for self-renewal of these HSPCs.

In summary, we demonstrated that Lhx2 maintains the un-differentiated state of human iPSC-derived HSPCs and suppresses their proliferation (Figure 5F). More importantly, Lhx2TAD stimulated self-renewal of human iPSC-derived HSPCs and this effect was promoted by UM171 (Figure 5F). Thus, we successfully amplified human iPSC-derived HSPCs *ex vivo* by modifying Lhx2. Recently, macrophages expressing chimeric antigen receptor are expected as cancer immunotherapy tools. Therefore, one of future directions of this study is utilization of iPSC-HSPCs obtained by Lhx2TAD as scalable sources of macrophages. On the other hand, current limitations of Lhx2TAD-transdused HSPCs are that *in vivo* hematopoietic repopulation ability was not observed in the transplanted mice, and that *in vitro* hematopoietic differentiation potentials other than myeloid lineages have not been demonstrated yet. Nevertheless, we believe that our study will make a significant contribution not only for understanding self-renewal and bone marrow repopulation ability of human iPSC-derived HSCs, but also for *ex vivo* production of human iPSC-derived immune cells.

## Data Availability

The datasets presented in this study can be found in online repositories. The names of the repository/repositories and accession number(s) can be found below: https://www.ncbi.nlm.nih.gov/, GSE44778; https://www.ncbi.nlm.nih.gov/, GSE273716.
