# Supplementary material for: Modification of Lhx2 activity for ex vivo amplification of human iPSC-derived hematopoietic stem/progenitor cells
Source: Front Cell Dev Biol. 2024 Oct 15;12:1482989. doi: 10.3389/fcell.2024.1482989 (PMC11518812; doi:10.3389/fcell.2024.1482989)
Supplement: Supplementary file 1 [file DataSheet1.PDF]

Supplemental information

1 Supplemental Materials and Methods

1.1 *In vitro* differentiation of human iPSCs by organoid system.

Single cell suspension of human iPSCs (5 – 10 x 10^4 cells) was seeded onto a conventional 60 mm tissue culture dish with 10 µM Y-27632 (Wako) and 1 µL/mL iMatrix-511 (Nippi) in mTeSR plus medium (StemCell Technologies). Two days later, medium was changed to mTeSR plus with 25 ng/mL BMP4 (Peprotech) and 7 µM CHIR99021 (Tocris) (day 0). On day 2, the cells were harvested and dispensed into low-attachment U-bottom 96-well plate (Greiner). The cells were seeded at 10,000 – 30,000 cells per wells in 0.1 mL mTeSR plus with 40 ng/mL VEGF (Peprotech) and 10 µM SB431542 (Wako). The plates were centrifuged (1,500 rpm, 5 min, 25˚C) and cultured for 3 days (day 2 to 5) to make embryoid bodies (EBs). On day 5, the resultant EBs were transferred onto gelatinized 48 well plate (Greiner, single EB per well) with 50 µL culture medium into 0.2 mL StemLineII (Sigma) with 40 ng/mL VEGF, 50 ng/mL IL-3 (Peprotech), and 50 ng/mL SCF (Peprotech) and cultured for 3 days (day 5 to 8). On day 8, the medium was replaced to 0.2 mL StemLineII with 40 ng/mL VEGF, 50 ng/mL IL-3, and 50 ng/mL SCF and further cultured for 3 days (day 8 to 11). On day 11, floating cells and adherent cells were recovered, large cell debris was removed by cell strainer and used for lentiviral vector transduction. The cells were then cultured in StemLine II with 50 ng/mL IL-3, 50 ng/mL IL-6 (Peprotech), and 50 ng/mL SCF.

1.2 Plasmid construction.

pMY-FLAG-Lhx2TAD-IRES-EGFP
The C-terminal region (nucleotides 1 – 987; amino acid 1 – 329) of the mouse Lhx2 cDNA was deleted by PCR. Then, DNA sequences encoding FLAG and VP16 TAD were inserted into 5' end and 3' end of above Lhx2 mutant, respectively. The resultant mutant, FLAG-Lhx2TAD, was sub-cloned into pMY-IRES-EGFP.

p6 x LBS-Luc
DNA oligonucleotide, cgg TAATTAGCTAATTA cgg (DNA sequences in lowercase were stuffers and the underlined sequences were Lhx2 binding sequence; LBS), was synthesized and six LBSs were inserted in tandem into pGL3-promoter plasmid (Promega).

pHR-IRES-ZsGreen1
The IRES-ZsGreen1 fragment was amplified from pLVSIN-EF1a-IRES-ZsGreen1 (Clontech) by PCR and inserted into a lentiviral vector, pHR (Addgene).

pHR-FLAG-Lhx2-IRES-ZsGreen1 & pHR-FLAG-Lhx2TAD-IRES-ZsGreen1
Human Lhx2 cDNA was purchased from Origene. DNA sequence encoding FLAG was inserted into 5' end of human Lhx2 by PCR. The resultant FLAG-Lhx2 fragment was inserted into pHR-IRES-ZsGreen1. Human Lhx2TAD was constructed by connecting VP16 TAD to 3' end of human Lhx2 C-terminal deletant. All sequences were confirmed by DNA sequence analyses.
Single cell suspension of human iPSCs (5 – 10 x 10^4 cells) was seeded onto a conventional 60 mm tissue culture dish with 10 µM Y-27632 (Wako) and 1 µL/mL iMatrix-511 (Nippi) in mTeSR plus

medium (StemCell Technologies). Two days later, medium was changed to mTeSR plus with 25 ng/mL

2. Supplemental Figures

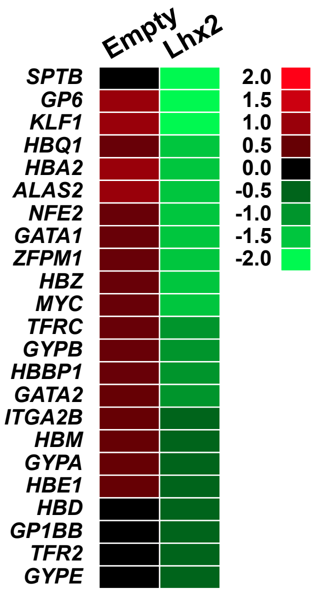

Supplemental Figure S1. Microarray analysis of K562 cells.
Heat map of erythroid and platelet-related genes. K562 cells transduced with empty vector (empty) and with Lhx2 (Lhx2) were compared.

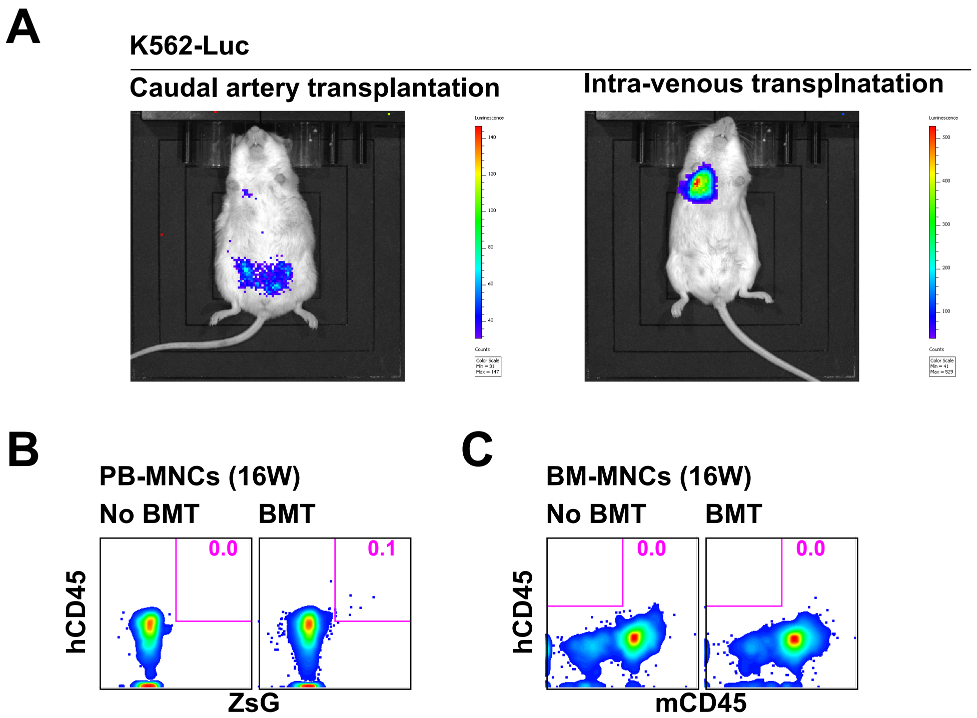

Supplemental Figure S2. Transplantation of Lhx2TAD-transduced cells.
A: Transplantation of K562 cells expressing luciferase (K562-Luc) into NSC mice via caudal artery or intra-venous. B, C: FACS analyses of recipient mice (BMT) and control mice (No BMT). Lhx2TAD was transduced on day 11 and whole cells on day 14 were transplanted into irradiated NSG mice via caudal artery. The peripheral blood mononuclear cells (PB-MNCs; B) and bone marrow mononuclear cells (BM-MNCs; C) were analyzed 16 weeks after transplantation. Representative data were shown. hCD45 = anti-human CD45 antibody. mCD45 = anti-mouse CD45 antibody.

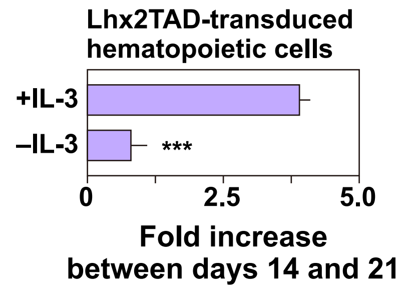

Supplemental Figure S3. Requirement of IL-3 for *ex vivo* expansion of transplantation of Lhx2TAD-transduced hematopoietic cells.
Fold increase of Lhx2TAD-transduced hematopoietic cells between days 14 and 21. These cells were cultured with IL-3, IL-6 and SCF (+IL-3) or IL-6 and SCF (–IL-3). Mean values with standard deviations are shown (n = 3). ***$p < 0.0005$ by the Student's t-test.

2 Supplemental Table S1

Antibodies used for FACS

Name	Reactivity	Clone	Conjugate	Company
Tie-2	mouse	TEK4	PE	Biolegend
c-Kit	mouse	2B8	PE/Cy7	Biolegend
Lin *1	mouse		biotin	Miltenyi Biotec
Sca-1	mouse	D7	APC	Biolegend
TIE-2	human	33.1	PE	Biolegend
CD34	human	581	PE/Cy7	Biolegend
CD43	human	CD43-10G7	APC	Biolegend
CD11B	human	ICRF44	APC	Biolegend
LIN *2	human		APC	Biolegend
CD45	human	HI30	BV421	Biolegend
CD38	human	HIT2	PE	Biolegend
CD90	human	5E10	AF647	Biolegend

*1: content of Lin cocktail
- CD5 (clone 53-7.3)
- CD11b (clone M1/70)
- B220 (clone RA3-6B2)
- Gr-1 (clone RB6-8C5)
- Ter-119 (clone Ter-119)

*2: content of LIN cocktail
- CD3 (clone SK3)
- CD14 (clone HCD14)
- CD16 (clone 3G8)
- CD19 (clone HIB19)
- CD20 (clone 2H3)
